# Supplementary material for: Zn2+ Aggravates Tau Aggregation and Neurotoxicity
Source: Int J Mol Sci. 2019 Jan 23;20(3):487. doi: 10.3390/ijms20030487 (PMC6387307; doi:10.3390/ijms20030487)
Supplement: Supplementary file 1 [file ijms-20-00487-s001.pdf]

Supporting Information

Zn^{2+} Aggravates Tau Aggregation and Neurotoxicity

by Xuexia Li, Xiubo Du, and Jiazuan Ni

Isothermal titration calorimetry (ITC).

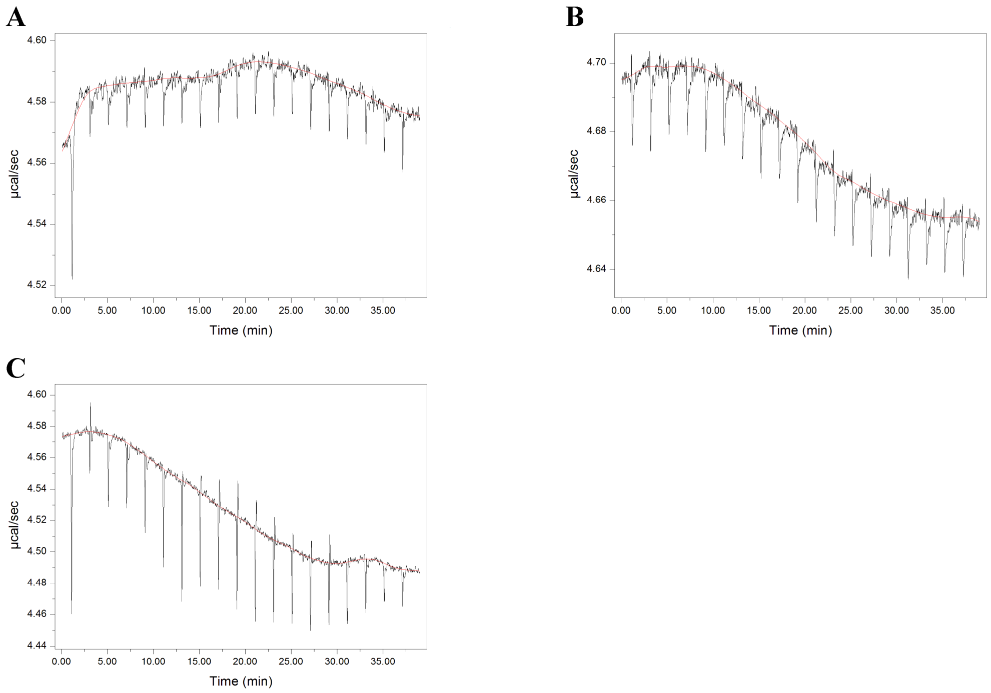

Figure S1. Properties of cations binding to tau-R3. (**A**) (**B**) and (**C**) ITC profiles for the binding of Mn^{2+}, Mo^{5+} or Fe^{3+} to Tau-R3, respectively.
